# *Glaphyrus festivus* Ménétriés, 1836 and related species in Turkey and Armenia (Coleoptera, Glaphyridae)

**DOI:** 10.3897/zookeys.1269.175618

**Published:** 2026-02-13

**Authors:** T. Ghrejyan, M. Kalashian, G. Sabatinelli, A. Frolov

**Affiliations:** 1 Institute of Zoology, Scientific Center of Zoology and Hydroecology, Yerevan, Armenia Institute of Zoology, Scientific Center of Zoology and Hydroecology Yerevan Armenia; 2 Museum d’histoire Naturelle de Genève, Genève, Switzerland Museum d’histoire Naturelle de Genève Genève Switzerland; 3 Zoological Institute RAS, Saint Petersburg, Russia Zoological Institute RAS Saint Petersburg Russia

**Keywords:** Asia Minor, bumble bee scarab beetles, lectotype designation, new species, taxonomy, Transcaucasia

## Abstract

The identity of *Glaphyrus festivus* Ménétriés, 1836, is evaluated in the Turkish and Armenian populations. Two new species, *G. neglectus* Ghrejan, Kalashian & Sabatinelli, **sp. nov**. from Armenia and eastern Turkey, and *G. ciliciensis* Ghrejan, Kalashian & Sabatinelli, **sp. nov**. from southern Turkey, are described and illustrated. A lectotype for *G. festivus* Ménétriés, 1836, is designated.

## Introduction

Édouard Ménétriés (1836) briefly described *Glaphyrus festivus* among other insects from the neighbourhoods of Constantinople (now Istanbul, Turkey). Later, Ménétriés (1839) provided a more detailed description and illustrated the species. Subsequent authors reported *G. festivus* from vast parts of West Asia. Von Harold (1869) recorded it from “Kleinasien; Erzerum, Anatolien”. Champenois (1903) expanded the distribution to include “Erivan (Korb), Asie Mineure: ‘Trebizonde’; Bitlis, Erzeroum, Bimbogha Dagh, Amasia, Perse: Fiaut-Karoun, Hamadan.” In the same year, Reitter (1903) reported *G. festivus* from “Erivan, Kleinasien, Anatolien, Kurdistan”. Zaitzev (1923), in addition to Yerevan, reported *G. festivus* from Eilar (now Abovyan, in Kotayk Province, Armenia), from Nakhichevan (Ordubad), and from Iran (West Azerbaijan: Nejchalon). Winkler (1929) did not specifically mention Armenia, likely including it under the broader context of Asia Minor and Iran. Medvedev (1960) reported the species from southern Armenia, Nakhichevan, as well as from eastern Turkey (Kurdistan), northern and northwestern Iran (Khoj, Luristan), and northern Iraq (Mossul), but did not mention west Turkey (Istanbul), the original locus of description. Iablokoff-Khnzorian (1967) reported *G. festivus* from Armenia, Nakhichevan, Asia Minor, and Iranian Azerbaijan. Baraud (1992) mistakenly cited the type locality as “Arménie (Erivan)” and reported the species from “Anatolie, Syrie, Iran.” Nikodým and Král (1998) reported the species from Iran (Lorestan). Nikodým and Keith (2007) indicated *G. festivus* from “Transcaucasus, Iran, Syria,” without mentioning Turkey. In both editions of the Catalogue of Palaearctic Coleoptera (Nikodým and Bezděk 2006, 2016), the distribution of this species is given as Azerbaijan, Armenia, Iran, and Turkey. Finally, Polat et al. (2017) reported the species from several provinces of Turkey (Adana, Erzincan, Erzurum, Kayseri), and Ghahari and Nikodým (2018) from west Azerbaijan.

The study of extensive material accumulated in the museums listed below under the name *G. festivus* shows that there is a complex of three closely related species, two of which are described below.

## Material and methods

Approximately 600 specimens of the *Glaphyrus festivus* complex from institutional and private collections were examined for this study.

The following abbreviations are used in the text to indicate the depositary of specimens:

**AKCM** Anton Kozloz personal collection, Moscow, Russia;

**COMP** Olivier Montreuil collection, Paris, France;

**DCCE** David Carlson personal collection, El Dorado Hills, California, USA;

**GSCG** Guido Sabatinelli personal collection, Prévessin, France;

**IZAY** Institute of Zoology, Scientific Center of Zoology and Hydroecology, Yerevan, Armenia;

**MHNG** Muséum d’Histoire Naturelle, Geneve, Switzerland;

**MKCY** Mark Kalashian research collection, Yerevan, Armenia;

**MNHN** Muséum National d’Histoire Naturelle, Paris, France;

**MNCP** Milan Nikodým personal collection, Roztoky u Praha, Czech Republic;

**MZHF** Finnish Museum of Natural History, Helsinki, Finland;

**NMPC** National Museum, Prague, Czech Republic;

**TGCY** Tigran Ghrejyan research collection, Yerevan, Armenia;

**ZIN** Zoological Institute RAS (St Petersburg, Russia);

In the citations of labels, current geographical names are provided in square brackets ([]) when necessary. The material was processed using traditional entomological methods. Male genitalia were extracted and prepared using standard techniques, then placed in a 12% water solution of KOH for a period of time, cleaned of membranous external structures, and washed with distilled water. The preparation of endophalli was done according to Kasatkin (2006) with minor modifications. Observations were made using Micromed MC-2 Zoom and MBS-10 stereomicroscopes. Measurements were taken with an ocular micrometre; body length was measured from the anterior margin of the clypeus to the elytral apex. Photographs were taken with a Canon EOS 800D digital camera, equipped with a Canon MP-E65 mm f/2.8 1–5x and LAOWA 100 mm f/2.8 Ultra Macro APO lenses, and mounted on a Stack Shot Macro Rail package (Cognisys Inc.). Photo stacking was performed using Helicon Focus Pro software.

## Taxonomic account

### Order Coleoptera Linnaeus, 1758

**Suborder Polyphaga Emery, 1886**

**Superfamily Scarabaeoidea Latreille, 1802**

**Family Glaphyridae Macleay, 1819**

**Subfamily Glaphyrinae Macleay, 1819**

**Genus *Glaphyrus* Latreille, 1802**

**Subgenus *Glaphyrus* Latreille, 1802**

The species under consideration can be easily distinguished from most of species of the subgenus *Glaphyrus* by mandibles which lack a tooth on the dorsal edge (in other species it bears more or less developed tooth – see Fig. 43) and by an arcuate incision between two apical teeth of the protibiae (other *Glaphyrus* species have an acutely angulate protibial incision). In addition to the species considered herein, these characters exhibit some similarity with *G. luristanus* Reitter, 1903, *G. calvaster* Zaitzev, 1923 and the recently described *G. sabatinellii* Ghrejyan, Kalashian & Nikodým, 2025. *Glaphyrus luristanus* differs by having sparser and somewhat finer pronotal punctuation, a sparser row of scale-like setae on the edge of the mesotibiae, by peculiarities of the setation of anterior angles of pronotum, which consists of row of dense rather tick brownish setae, and by the hind trochanter nearly straight cut laterally. The other two species can be easily distinguished by more slender hind femora in males, which in *G. sabatinellii* are 2.2 × and in *G. calvaster* ~ 2.6 × as wide as long. The diagnostic characters of the species considered herein are included in Table 1.

**Table 1.** Summary of diagnostic characters between the species of the *Glaphyrus festivus* complex.

Species	*Glaphyrus festivus*	*Glaphyrus ciliciensis* sp. nov.	*Glaphyrus neglectus* sp. nov.
Characters			
Integument colouration	Green, violet, blue with blue or greenish shine, rarely nearly black with violet tint	Green, violet, blue with blue or greenish shine, rarely black	Black, rarely with very indistinct violet, cobalt-blue of greenish tint
Elytral stripes of short whitish-yellowish hairs	Only traces in male, slightly visible apically in female	Only traces in male, slightly visible apically in female	Clearly visible in both sexes, more developed in female
Pronotal lateral margin	Very slightly sinuate in basal 1/2 (Fig. 17)	Regularly arcuate (Fig. 19)	Straight in basal 2/3 (Fig. 21)
Pronotal punctuation	Rough and dense, basal “mirrors” relatively large (Fig. 17)	Fine and scattered, basal mirrors relatively large (Fig. 19)	Rough and dense, basal mirrors relatively small (Fig. 21)
Pronotal pubescence	Relatively long and sparse	Relatively short and dense	Relatively short and dense
Elytral sides in lateral view	Distinctly sinuate (Fig. 7)	Moderately sinuate (Fig. 8)	Very slightly sinuate (Fig. 9)
Apical edge of elytra	Slightly convex, without distinct groove, with row of sparse, dark, thick, setae (Fig. 37)	Flattened, without distinct groove, with row of sparse dark, thick, setae (Fig. 39)	With a clear groove separating convex edge, with row of dense, dark, thick, setae (Fig. 41)
Hind femora in male	Relatively thick (Fig. 31)	Relatively narrow (Fig. 33)	Relatively tick (Fig. 35)
Mesotibia in male	With row of punctures dorsally bearing short setae reaching ~ 2/3 of tibia length (Fig. 25)	With row of punctures dorsally bearing short setae reaching ~ 3/5 of tibia length (Fig. 26)	With row of punctures dorsally bearing short setae reaching ~ 3/4 of tibia length (Fig. 27)
Paramere	Intermediate width (Fig. 44)	Narrow (Fig. 46)	Wide (Fig. 48)
Endophallus	Fig. 45	Fig. 47	Fig. 49

#### Glaphyrus (Glaphyrus) festivus

Taxon classificationAnimaliaColeopteraGlaphyridae

Ménétriés, 1836

3D918C87-9EFC-5C28-9BF0-7D66FDA746A8

Figs 1, 4, 7, 10, 11, 16, 17, 22, 25, 28, 31, 32, 37, 38, 44, 45, 51, 55

Glaphyrus (Glaphyrus) festivus Ménétriés, 1836: 150; Harold 1869: 434 (pars); Champenois 1903: 139, 146 (pars); Reitter 1903: 128 (pars); Baraud 1992: 405, 407 (pars); Nikodým and Bezděk 2006: 100 (pars); Nikodym and Keith 2007: 3 (pars); Nikodým and Bezděk 2016: 95 (pars); Polat A. et al. 2017: 1509 (pars).
Glaphyrus rufipennis : Nikodým and Bezděk 2006: 100; Nikodým and Bezděk 2016: 95.

##### Type material.

***Lectotype*. Turkey** • ♀ Turcia; 241-2 (h); festivus Mén. (h); ZIN (Fig. 4).

##### Material examined.

**Turkey** • 1 ♂, Constantinople; MNHN; • 4 ♂♂, 3 ♀♀, Angora; MNHN; 1 ♂, 2 ♀♀, Asia Minor; ZIN • 1 ♂, Erzurum; ZIN • ♀; “Turcia” ZIN. 6 ♂♂, 2 ♀♀, [Turkey] Ankara; Golbashi; 18.VI.1970; MHNG; GSCG • 1 ♂; Angora [Ankara]; Escherich, 1895; GSCG; • 1 ♂, Erzincan; 12.VII.1972; MNHN; • 3 ♂♂, 2 ♀♀, Turkei; Nigde; Yesilhisar-Camardi; 07.VI.1966; Petrovitz leg.; MNHN; • 1 ♂, Bimbogha Dagh; Escalera leg.; MNHN; • 8 ♂♂, 5 ♀♀; Erzincan prov.; 39°45'N, 40°19'E; 1439 m; 7.VII.2018; G. Sabatinelli leg.; GSCG • 1♂, 1♀, Anatolien; Gevas; Karabety Pass; VII.1987; Heinz leg.; GSCG • 1 ♂; Elazig; 28.VI.1950; 12 ♂♂, 7 ♀♀, Malatya vil.; Karahan gecidi; Kürecik; 1785 m; 38°29'N, 37°48'E; 1785 m; 30.VI.2000; G. Sabatinelli leg.; GSCG • 16 ♂♂, 11 ♀♀, Malatya prov.; 50 km N. Darende; 1195 m; 38°40'N, 37°26'E; 29.VI.2000; G. Sabatinelli leg.; GSCG • 5 ♂♂, 5 ♀♀; Malatya vil.; Resadiye gec.; VI.2002; Malmusi leg.; GSCG • 1♂; Erzincan prov.; Tercan; 1493 m; 7.VII.2018; G. Sabatinelli leg.; GSCG • 3 ♂, 2 ♀; Sivas vil.; Gürün; Gökpınar; 1465 m; 29.VI.2000; G. Sabatinelli leg.; GSCG) • 1 ♂; Malatya; 4.7.98; Farbiak leg.; NMPC • 1 ♂; Sivas; NMPC • 1 ♂; Anatolia; NMPC • 1 ♀; Asia Minor; NMPC • 2 ♂♂; Asia Minor; Reitter leg.; NMPC • 3 ♂♂, 2 ♀♀; Anatolia; Korčnsky leg.; NMPC • 4 ♂♂, 3 ♀♀; Angora;, V.M. Duchon leg.; NMPC • 1 ♂; Angora; Escherich; 1895; NMPC • 2 ♂♂,1 ♀; Tuz Golu; J. Hájek leg.; NMPC • 1 ♀; Kayseri; Sultanshah; 1200 m;15.VI.62; Guichard & Harvey leg.; NHMUK.

##### Re-description.

**Figures 1–6.**
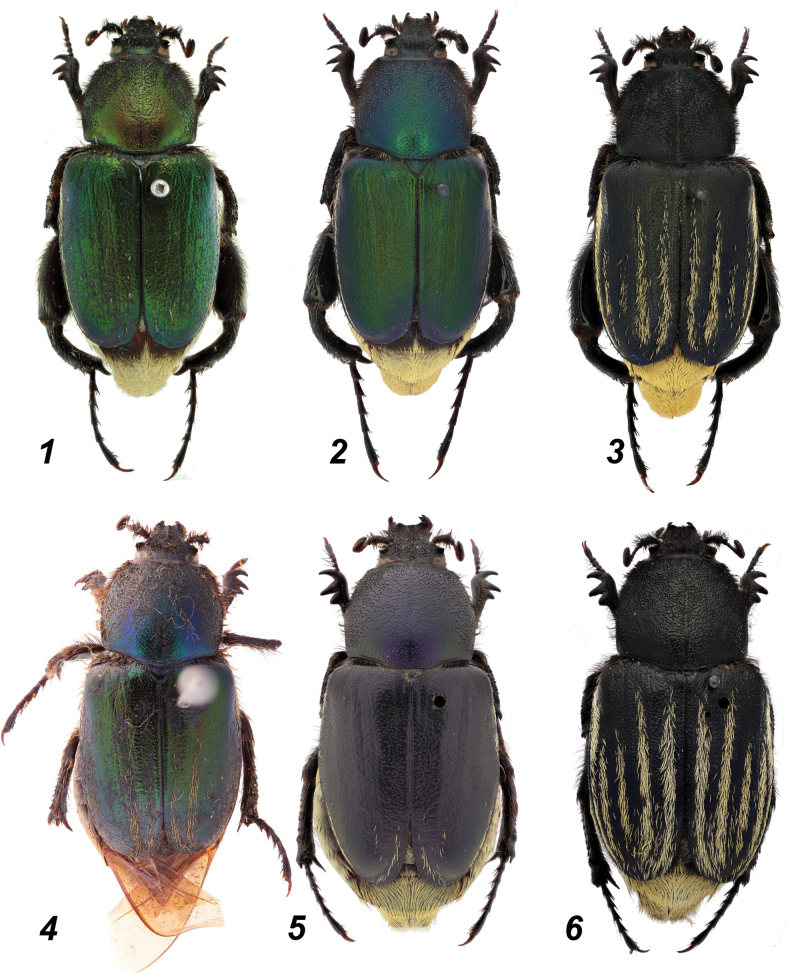
*Glaphyrus* spp., habitus. **1**. *G. festivus* Ménétriés, 1836, Turkey, Ankara; **2, 5**. *G. ciliciensis* sp. nov. (paratypes); **4**. *G. festivus* Ménétriés, 1836 (lectotype); **3, 6**. *G. neglectus* sp. nov. (holotype and paratype). Males (**1–3**), females (**4–6**).

**Figures 7–9.**
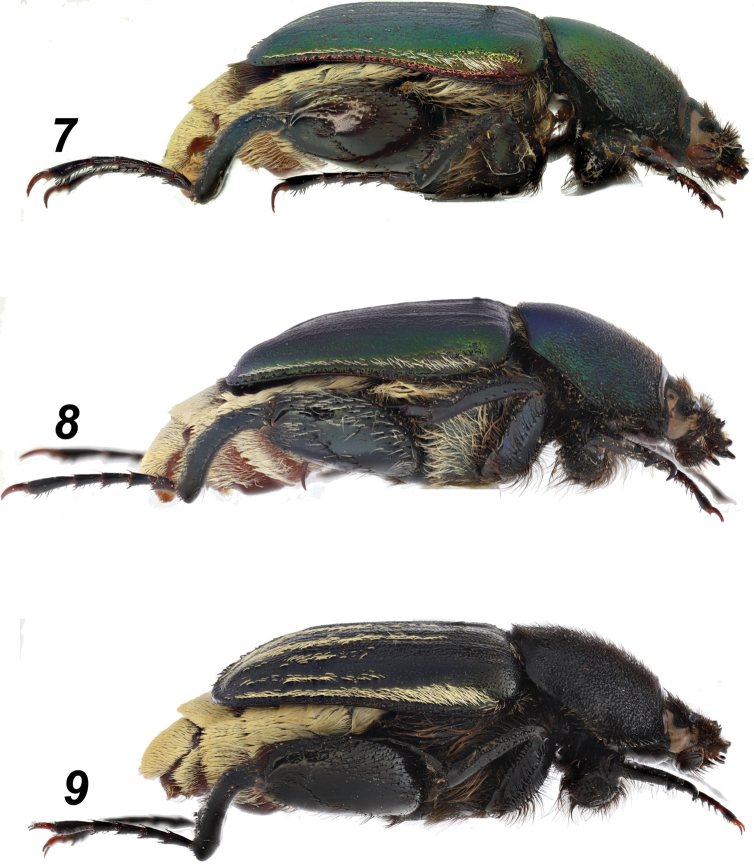
*Glaphyrus* spp., males, lateral view. **7**. *G. festivus* Ménétriés, 1836, Turkey, Ankara; **8**. *G. ciliciensis* sp. nov., holotype; **9**. *G. neglectus* sp. nov., holotype.

**Male. *Body*** (Figs 1, 7) rather large and robust, shiny, dark metallic green, sometimes bluish or violet, rarely nearly black with a fine violet reflection. Length: 14.7–16.8 mm, width: 7.2–8.6 mm.

***Head*** (Fig. 16). Anterior margin of clypeus with widely rounded protruding anterior angles and a sharp tooth pointing upwards medially. Integument covered with dense, irregular, rough punctures, and sparse, short brownish hairs. Frons with similar punctures, with rather dense and long blackish setae posteriorly. Fronto-clypeal suture somewhat obscured by the sculpture but remains visible. First antennomere large, distinctly incurved, and swollen distally, with dense, long dark hairs and short, thick brownish setae. Second antennomere ~3 × shorter, swollen, with dense dark hairs. Antennomeres 3–7 each have a single short, delicate seta, and the antennal club has a single short oblique seta, shorter than the setae on antennomeres 3–7 (Fig. 16). Dorsal margin of mandibles nearly regularly rounded, without a tooth (Fig. 38). Last segment of maxillary palps distinctly narrowed distally, with a truncate apex.

**Figures 10–15.**
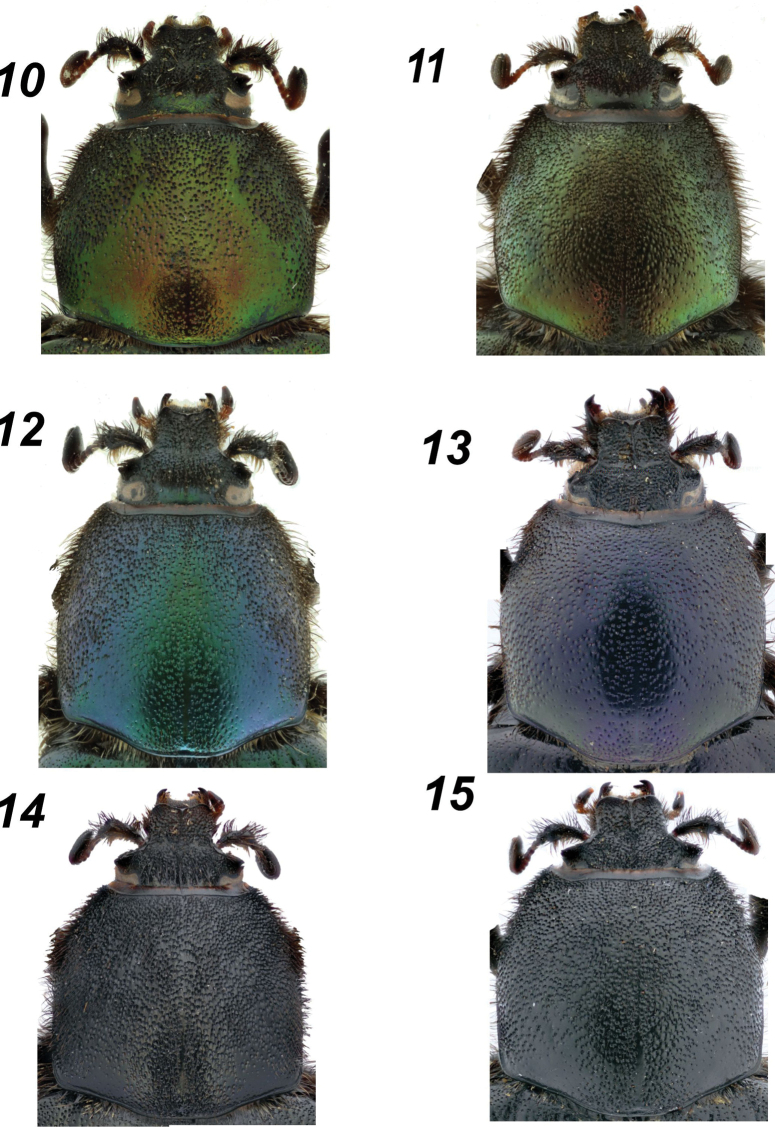
*Glaphyrus* spp., head and pronotum. **10, 11**. *G. festivus* Ménétriés, 1836, Turkey, Ankara, male and female); **12, 13**. *G. ciliciensis* sp. nov. (holotype, male and paratype, female); **14, 15**. *G. neglectus* sp. nov. (holotype, male and paratype, female).

**Figures 16–21.**
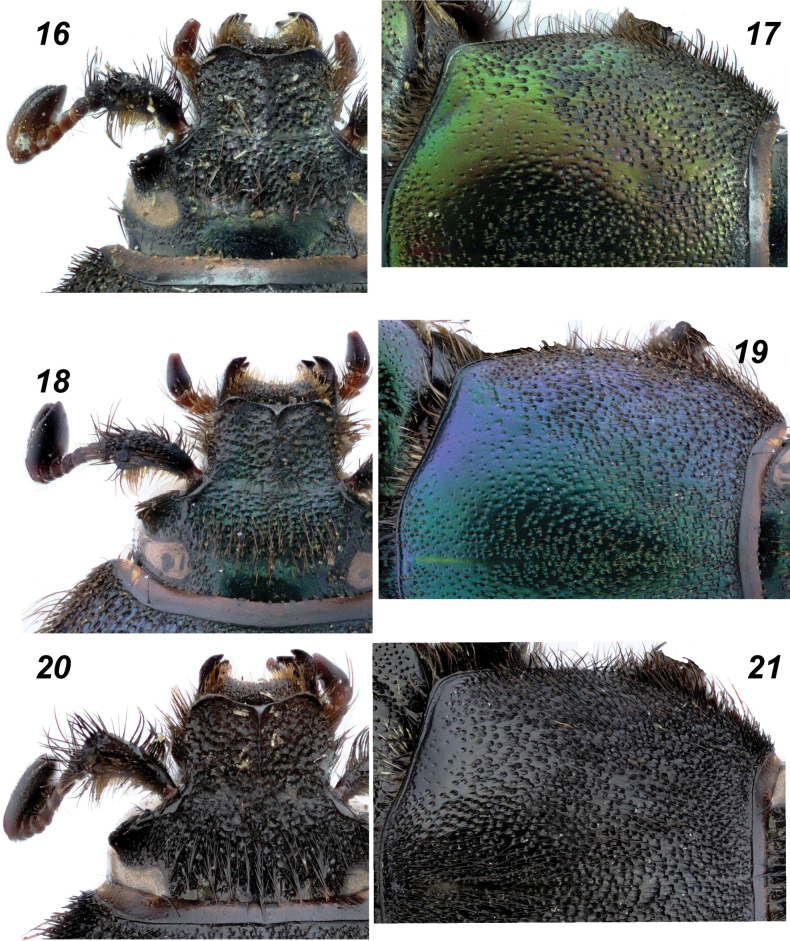
*Glaphyrus* spp., males, details. **16, 17**. *G. festivus* Ménétriés, 1836, Turkey, Ankara; **18, 19**. *G. ciliciensis* sp. nov. (holotype); **20, 21**. *G. neglectus* (holotype). Head (**16, 18, 20**), pronotum (**17, 19, 21**).

***Pronotum*** (Figs 10, 17) rather convex, with sides arcuate, moderately convergent anteriorly, and very finely sinuate and slightly convergent toward obtusely rounded posterior angles. Anterior margin finely bisinuate with sharp angles, featuring a distinctly detached shiny edging. Posterior margin bisinuate with a protruded medial lobe, with shiny edging separated by a distinct deep groove, which is shortly continued forward along the lateral margins. Disc with dense, rough, partly contiguous punctures, though the punctation is somewhat finer than in *Glaphyrus neglectus* sp. nov., pair of small, roughly triangular, nearly glabrous areas (“mirrors”) larger. Integument without microreticulation, thus appearing rather shiny, covered with dense, dark brownish hairs directed mainly laterally and backwards, without glabrous areas. Anterior angles with a brush of dense, short, thick dark brown setae.

***Scutellum*** small, with small, rather single punctures anteriorly, becoming smoother posteriorly.

***Elytra*** moderately convex, with sides widely arcuate and apices rounded but slightly angular. The “striped” pattern on the elytra is very faint, with only traces of stripes or single yellowish hairs; a lateral stripe runs from the humeral tubercle, almost reaching the elytral apex. Surface densely punctured medially with rough punctures, while the remaining area with sparse, small, irregular punctures, without microreticulation thus shiny. Integument with sparse long, irregular, twisted longitudinal wrinkles. Lateral margins with a distinct groove, almost completely obscured apically. In lateral view, the lateral margins are distinctly sinuous (Fig. 7). Apical edge of elytra slightly convex, without distinct groove, with row of sparse, dark, thick, setae (Fig. 37).

***Pygidium and propygidium*** surfaces covered with dense, short yellowish setae, completely hiding the small and dense punctate surface.

***Thorax*** densely punctate with rough punctures and dense, long, curved hairs. On the prosternum, the hairs are mostly dark brown, sometimes mixed with a few yellowish ones, while on the metasternum, they are predominantly yellowish, with the pubescence medially sparser and shorter, but denser laterally, sometimes hiding the surface.

**Figures 22–24.**
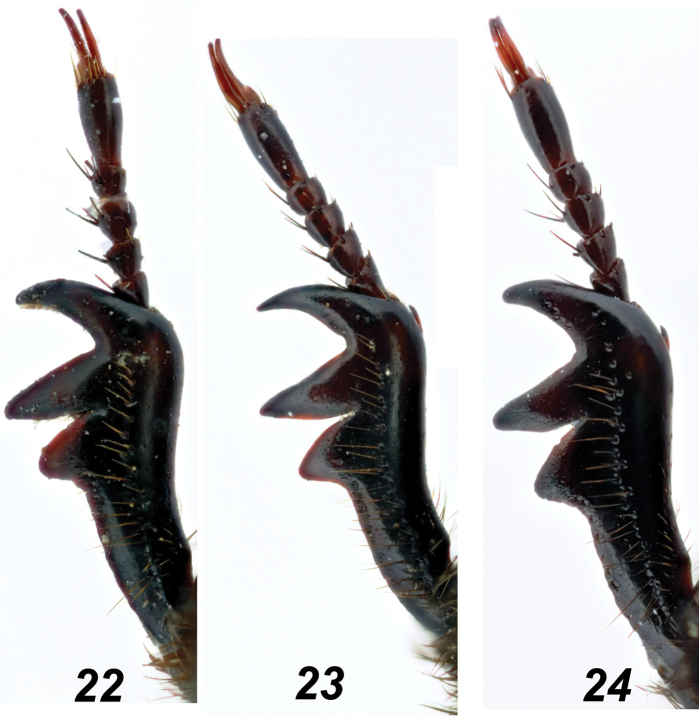
*Glaphyrus* spp., males, fore tibiae. **22**. *G. festivus* Ménétriés, 1836, Turkey, Ankara; **23**. *G. ciliciensis* sp. nov. (holotype); **24**. *G. neglectus* sp. nov. (holotype).

**Figures 25–30.**
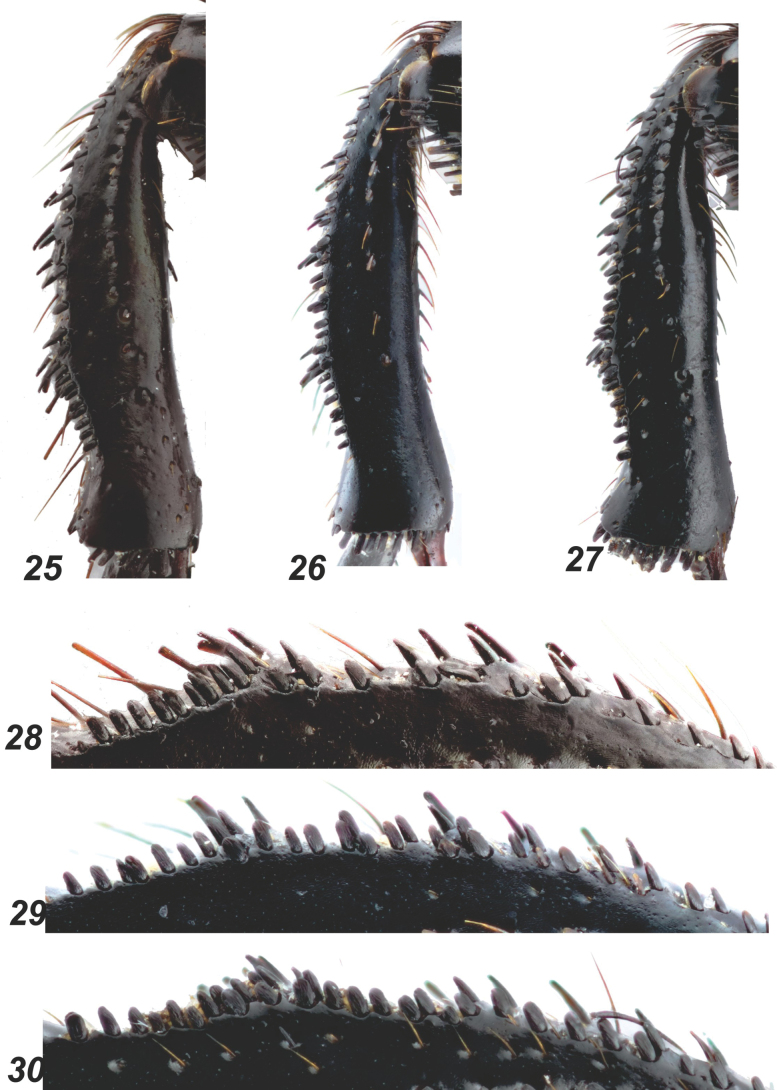
*Glaphyrus* spp., males, mesotibiae. **25, 28**. *G. festivus* Ménétriés, 1836, Turkey, Ankara; **26, 29**. *G. ciliciensis* sp. nov., holotype; **27, 30**. *G. neglectus* sp. nov., holotype.

**Figures 31–36.**
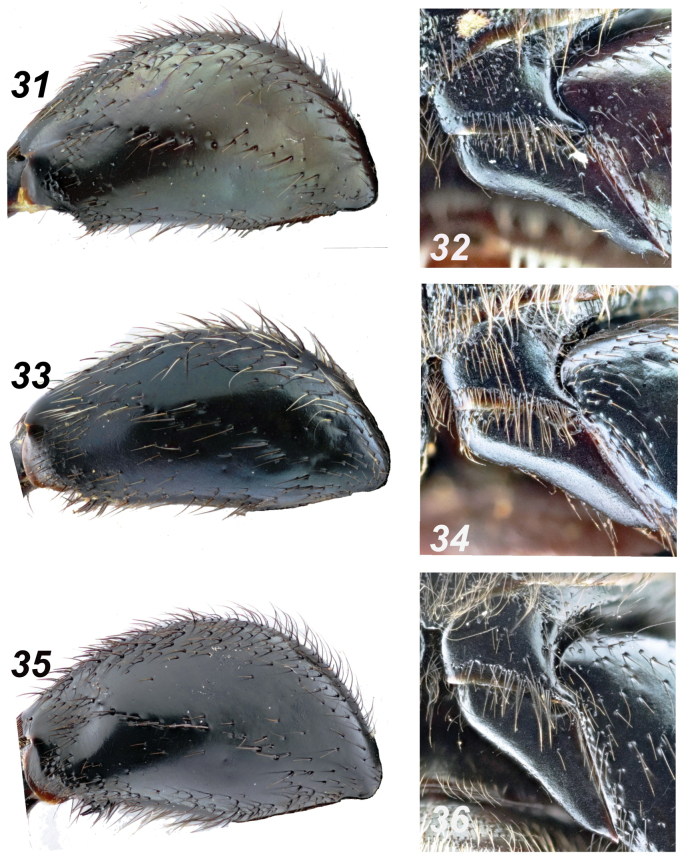
*Glaphyrus* spp., males, details. **31, 32**. *G. festivus* Ménétriés, 1836, Turkey, Ankara; **33, 34**. *G. ciliciensis* sp. nov., holotype; **35, 36**. *G. neglectus* sp. nov., holotype. Hind femora (**31, 33, 35**), trochanters (**32, 34, 36**).

***Legs***. Protibiae (Fig. 22) with three large lateral blunted teeth, the edge proximally is nearly straight, somewhat uneven. The incision between the two distal teeth is narrowly somewhat unevenly arcuate. The dorsal surface has a slightly uneven row of punctures bearing long brown hairs and edged with a rather indistinct groove. The row of punctures reaches approximately the level of the incision between the two distal teeth. Mesotibiae (Figs 25, 28) outer edge nearly regularly widely arcuate, with fine protrusion near distal 1/3 and with an irregular row of wide, flat, scale-like setae. Dorsal surface with a row of deep punctures bearing short dark hairs, these punctures close proximally, scattered distally, and reaching approximately the level of the distal 2/3 of the tibia. Metafemora (Fig. 31) thick, ~ 1.8 × as long as wide, with an irregular row of distinct rasp-shaped punctures along the middle, bearing short brownish setae; proximally and distally, row enlarges into areas with similar punctures, continuing along the anterior and posterior margins of the femur. Between the medial row and the punctate margins, the surface is nearly glabrous, with single small punctures. The large spur of the hind tibia is shorter than the first tarsomere, and the tarsi are rather slender. Trochanter laterally with a narrow tooth (Fig. 32).

***Abdomen*** integument with rather small and superficial punctures and long yellowish hairs that somewhat obscure the integument. Fifth ventrite with a distinctly elevated glabrous, hairless callosity, behind which is a row of thin, short, erect hairs situated adjacent to a rather wide, also glabrous and hairless, slightly convex stripe separating the callosity from the posterior margin of the ventrite.

***Aedeagus*** paramere as shown in Fig. 44, and endophallus as shown in Fig. 45.

**Figures 37–43.**
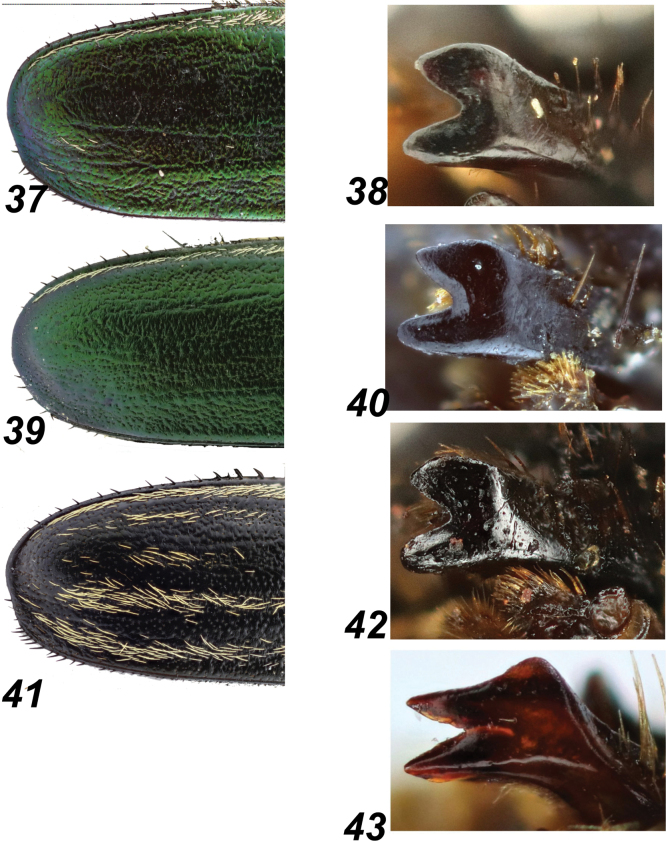
*Glaphyrus* spp., males, details. **37, 38**. G. festivus Ménétriés, 1836, Turkey Ankara; **39, 40**. *G. ciliciensis* sp, nov., holotype; **41, 42**. G. neglectus sp. Nov., holotype; **43**. *G. micans* (Faldermann, 1835), Armenia. Elytral apex (**37, 39, 41**), left mandible (**38, 40, 42, 43**).

**Figures 44–49.**
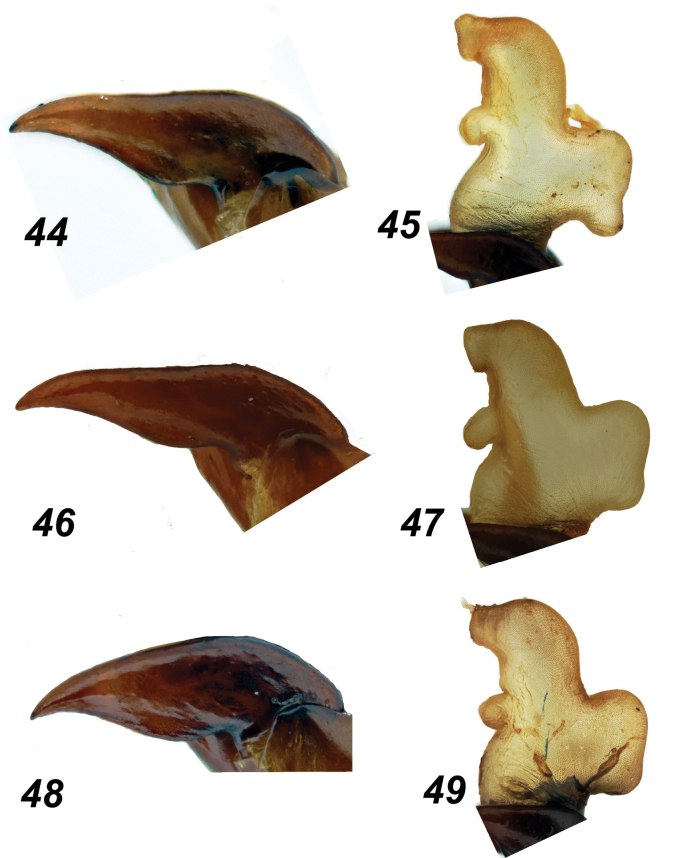
*Glaphyrus* spp., male genitalia. **44, 45**. *G. festivus* Ménétriés, 1836, Turkey, Ankara; **46, 47**. *G. ciliciensis* sp. nov., paratype; **48, 49**. *G. neglectus* sp. nov., paratype; paramere (**44, 46, 48**), endophallus (**45, 47, 49**).

**Figures 50–55.**
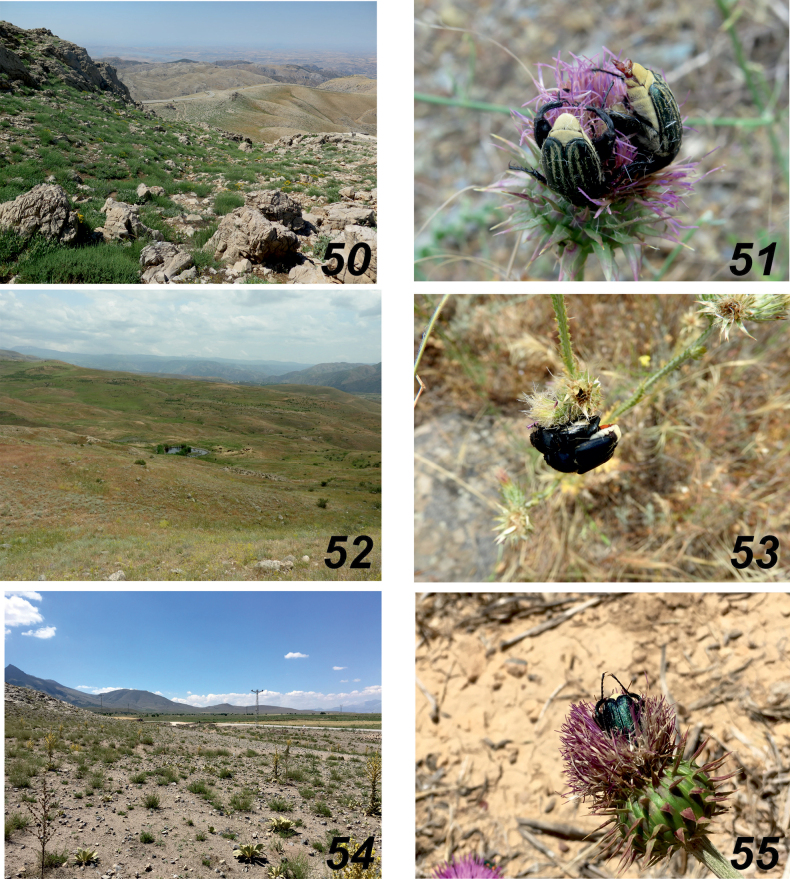
*Glaphyrus* spp. **50, 51**. *G. neglectus* sp. nov., Armenia, Hatsavan; **52, 53**. *G. ciliciensis* sp. nov., Karaman; **54, 55**. *G. festivus*, Ménétriés, 1836, Malatia. Habitat (**50, 52, 54**), feeding beetles (**52, 53, 55**).

**Female**. Body (Fig. 4) on average somewhat smaller than in males, length 13.5–15.0 mm, width 6.7–7.8 mm. Punctuation of pronotum somewhat denser but finer than in male, posterior “mirrors” somewhat smaller. Traces of striped elytral pattern visible in posterior portion of elytra. Hind legs shorter than in males, femora not enlarged.

##### Type locality.

In the original description of *G. festivus* the locality is stated as “environs de Constantinople” [= Istanbul]. Only one specimen of the species labelled “Constantinople” was found among more than 600 examined. It can be assumed that the old populations of this species are extinct.

##### Distribution.

**Figure 56.**
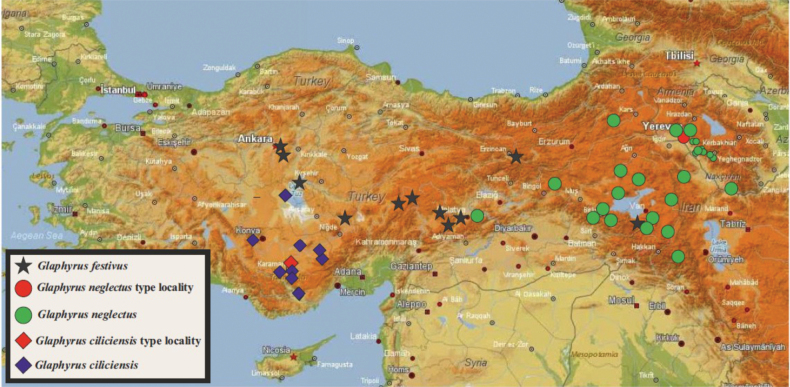
Distribution map of *Glaphyrus* species (map basis: https://nakarte.me/#m=6/35.84453/47.57080&l=T).

The species, as here defined, was found in most of north-western, central, and south-eastern Turkey, with records from the provinces of Ankara, Malatya, Sivas, Kayseri, and Erzincan, and from Istanbul (see map in Fig. 56).

##### Remarks.

Edouard Ménétriés was the first curator of entomological collections of the Zoological Museum of the Academy of Sciences (now ZIN). In 1830s, the Academy acquired a collection of insects from Wiedemann, who spent many years in Turkey. Ménétriés examined this material and published a note with short Latin diagnoses of a number of new species including *Glaphyrus festivus* among five scarab beetles species (Ménétriés 1836). From the diagnosis of *G. festivus*, it is unclear how many specimens Ménétriés did examine or their sex. However, the indication of the colour variation (“viridi- aut violaceo-aereus”) suggests that at least two specimens with different hues were examined. In a latter work (Ménétriés 1839), he repeated the Latin diagnosis of the species and supplemented it with an expanded French description, size range, and a colour illustration, but did not provide any specimen-specific information. In the ZIN collection, there is a series of specimens near the drawer bottom labelled with Ménétriés’ handwriting “festivus Mén”. Of these, there are five specimens bearing small labels with 4-digit numbers, a number from 1 to 5, and additional data “Turcia”, “Asia Minor”, “Erivan”, or “Ms. Ararat”. These labels were placed by August Morawitz, who became the curator after Ménétriés had retired. There are no records found explaining what the 4-digt numbers mean exactly, but apparently it was the first attempt to reorganize and catalogue the collection by Morawitz and the 4-digit number correspond to a species and the number after a hyphen to a specimen in a series. As Ménétriés did not mention any other regions except for Turkey, it is most probable that the description *G. festivus* was based the specimen (Fig. 4) which now bears the label “Turcia 5241 - 2”. Also, this specimen was mounted with expanded legs, which is characteristic for early insect collection preparations targeted for public display. Considering that the specimen agrees with Ménétriés’ original description, in size range and locality data, we believe that this specimen should be considered the syntype.

Considering the revealed complexity of the taxonomic structure of the species formerly referred to as *Glaphyrus festivus* Ménétriés, 1836, we deemed it necessary to clarify the application of the Ménétriés’ name by designating a lectotype. As the lectotype, we designate a female specimen (Fig. 4), which was most probably that illustrated by Ménétriés (1839: tab. I, fig. 8).

After the description of *Glaphyrus festivus*, subsequent authors attributed specimens collected from regions such as “Asia Minor,” “Transcaucasia,” “Russian Armenia,” “Kurdistan,” and others to Ménétriés’ species. In particular, the detailed description of *G. festivus* by Medvedev (1960) was most likely based on specimens of *G. neglectus* sp. nov. Additionally, Medvedev’s distribution did not even mention west Turkey, from where *G. festivus* was originally described. Baraud (1992) inexplicably listed “Arménie (Erivan)” as the type locality for *G. festivus*; in fact, all records of *G. festivus* from Transcaucasia, as well as from northeastern Turkey and northwestern Iran, most likely refer to the newly described *G. neglectus* sp. nov.

##### Biological notes.

The species inhabits dry steppes and semi-deserts and readily occupies ruderal habitats such as abandoned arable lands and overgrazed slopes, often overgrown with thorny Asteraceae (e.g., *Onopordum acanthium*, Onopordum cf. armenum, *Tragopogon* sp., *Carduus nutans*), where it feeds by burying itself in their inflorescences. The beetles are active from late June to mid-July (Figs 54, 55).

##### Variability.

Most specimens of this species are entirely greenish with a reddish, purplish, or bluish sheen, occasionally exhibiting a darker blue or violet hue. In some rare specimens, the elytra are reddish-brown and a fully black form is extremely rare. The elytra feature distinct longitudinal stripes of short whitish hairs. Discriminatory characters with related species are provided in Table 1.

#### Glaphyrus (Glaphyrus) ciliciensis

Taxon classificationAnimaliaColeopteraGlaphyridae

Ghrejan, Kalashian & Sabatinelli sp. nov.

25297B4B-A5C3-573A-8AD7-57FF9B1B33AE

https://zoobank.org/5F01E0BF-C837-4EB4-A4EB-317263419BD4

Figs 2, 5, 8, 12, 13, 18, 19, 23, 26, 29, 33, 34, 39, 40, 46, 47, 52, 53

Glaphyrus festivus Ménétriés, 1836: Harold 1869: 434 (pars); Champenois 1903: 139, 146 (pars); Reitter 1903: 128 (pars); Zaitzev 1923: 107, 114 (pars); Baraud 1992: 405, 407 (pars); Nikodým and Bezděk 2006: 100 (pars); Nikodym and Keith 2007: 3 (pars); Nikodým and Bezděk 2016: 95 (pars); Polat A. et al. 2017: 1509 (pars); Ghahari and Nikodým 2018: 1184 (pars).

##### Type material.

***Holotype*. Turkey** • ♂; Vil[ayet] Karaman; [10 km S Karaman]; Lale; 37°02'N, 33°16E; 1375 m; 11.VI.2016, G. Sabatinelli leg.; MHNG. ***Paratypes*. Turkey** • 12 ♂♂, 3 ♀♀RKEY eses; 4 allotype ♀, same data as for holotype; GSCG • 6 ♂♂, 2 ♀♀; Vil[ayet] Karaman; 1125 m; 7 km South Karaman; 37°06'N, 33°14'E; 13.VI.2013; G. Sabatinelli leg.; GSCG • 4 ♂♂, 2 ♀♀, Karaman; Hamzazindani; 15.VI.2015; O. Kochak leg.; GSCG • 1 ♂, 2 ♀♀; Vil[ayet] Karaman; 1110 m; Pinarbasi; 37°07'N, 33°06'E; 11.VI.2013; G. Sabatinelli leg.; GSCG • 2 ♂♂, 1 ♀; Vil[ayet] Konya; 10 km S Konya; 1030 m; 38°00'N, 33°57'E; 10.VI.2016; G. Sabatinelli leg.; GSCG • 1 ♂; Mut Yolum; 24.VI.2015; O. Kocak leg.; GSCG • 1 ♂; Cilicia; Taurus; Zw. Bor und Ciftehan; 8.VI.1966; GSCG • 2 ♂♂; Nigde; Taspinar, 10.VI.1966; GSCG • 1 ♀; Karaman; Gokge koyu; 5.VI.2016; O. Kochak leg.; GSCG • 1 ♀ Karaman; Karadag; May 2011; GSCG • 1 ♂, Aksaray; 06.VI.1991; G. Minet leg.; MNHN • 3 ♂♂ eKaraman; Derekoy; 17.VI.2015; O. Kochak leg.; GSCG; • 1 ♂, 2 ♀♀, Konya; MNHN; • 1 ♂; Tuz Golu; Jiří Hájek leg.; NMPC; GSCG • 1 ♂; Klein Asien; Konia; H. Holzchut; NMPC • 1 ♂; 20 km NNE Konya; Konya National Park; 18.VI.2009; M. Kalashian leg. MKCY • 2♂♂; Gaybi b.[is] Eregli Asia minor; Petrovitz-Ressl leg.; MNCP.

##### Description.

**Male. *Body*** (Fig. 2) rather large and robust, shiny, dark metallic green, sometimes bluish or violet, rarely nearly black with a fine violet reflection. Length: 14.6–16.9 mm (holotype 15.8), width: 7.1–8.5 mm (holotype 7.7 mm).

***Head*** (Fig. 18). Anterior margin of clypeus with widely rounded protruding anterior angles and a sharp tooth pointing upwards medially. Integument covered with dense, irregular, rough punctures, and sparse, short, brownish hairs. Frons with similar punctuation, with rather dense and long blackish setae posteriorly. Fronto-clypeal suture somewhat obscured by the sculpture but remains visible. First antennomere large, distinctly incurved, and swollen distally, with dense and long dark hairs and short, thick brownish setae. Second antennomere ~3 × shorter, swollen, with dense dark hairs. Antennomeres 3–7 each have a single short, delicate seta, and the antennal club has a single short oblique seta, shorter than the setae on antennomeres 3–7 (Fig. 18). Dorsal margin of mandibles nearly regularly rounded, without a tooth (Fig. 40). Last segment of maxillary palps distinctly narrowed distally, with a truncate apex.

***Pronotum*** (Figs 12, 19) rather convex, with sides arcuate, moderately convergent anteriorly, and slightly convergent toward obtusely rounded posterior angles. Anterior margin finely bisinuate with sharp angles, featuring a distinctly detached shiny edging. Posterior margin bisinuate with a protruded medial lobe, with shiny edging separated by a distinct deep groove, which is shortly continued forward along the lateral margins. Disc with dense, rough, partly contiguous punctures, though the punctuation is somewhat finer than in *Glaphyrus neglectus* sp. nov., and the basal “mirrors” larger. Integuments without microreticulation, thus appearing rather shiny, covered with dense dark brownish hairs directed mainly laterally and backwards, without glabrous areas. Anterior angles with a brush of dense, short, thick dark brown setae.

***Scutellum*** small, with small, rather single punctures anteriorly, becoming smoother towards the posterior.

***Elytra*** moderately convex, with sides widely arcuate and apices rounded but slightly angular. The “striped” pattern on the elytra is very faint, with only traces of stripes or single yellowish hairs; a lateral stripe runs from the humeral tubercle, almost reaching the elytral apex. Surface densely punctured medially with rough punctures, while the remaining area with sparse, small, irregular punctures, without microreticulation thus shiny. Integuments with sparse long, irregular, twisted longitudinal wrinkles. Lateral margins with a distinct groove, almost completely obscured apically (Fig. 39). In lateral view, the lateral margins are moderately sinuous (Fig. 8). Apical edge of elytra flattened, without distinct groove, with row of sparse dark, thick, setae (Fig. 39).

***Pygidium and propygidium*** surfaces covered with dense, short yellowish setae, completely hiding the small and dense punctuate surface.

***Thorax*** densely punctate with rough punctures and dense, long, curved hairs. On the prosternum, the hairs are mostly dark brown, sometimes mixed with a few yellowish ones, while on the metasternum, they are predominantly yellowish with the pubescence medially sparser and shorter, but denser laterally, sometimes hiding the surface.

***Legs***. Protibiae (Fig. 23) with three large lateral teeth, the edge is nearly straight proximally, somewhat uneven. The incision between the two distal teeth is narrowly arcuate. The dorsal surface has a slightly uneven row of punctures bearing long brown hairs and edged with a distinct groove. The row of punctures reaches approximately the level of the incision between the two distal teeth. Mesotibiae (Figs 26, 29) outer edge nearly regularly widely arcuate, with nearly indistinct protrusion near distal 1/3 and with an irregular row of wide, flat scale-like setae long the outer margin. Dorsal surface with a row of deep punctures bearing short dark hairs, these punctures are close proximally, scattered distally and reaching approximately the level of the distal 2/3 of the tibia. Metafemora (Fig. 33) slightly slenderer than in other sibling species, ~1.95 × as long as wide, with an indistinct row of distinct rasp-shaped punctures along the middle, bearing short whitish setae that are longer than in *G. neglectus* sp. nov.; proximally and distally, this row enlarges into areas with similar punctures, continuing along the anterior and posterior margins of the femur. Between the medial row and the punctate margins, the surface is nearly glabrous, with single small punctures. The large spur of the hind tibia is shorter than the first tarsomere, and the tarsi are rather slender. Trochanter laterally with a narrow tooth (Fig. 34).

***Abdomen*** integuments with rather small and superficial punctures and long yellowish hairs that somewhat obscure the integument. Fifth ventrite with a distinctly elevated, glabrous, hairless callosity, behind which is present a row of thin, short, erect hairs situated adjacent to a rather wide, also glabrous and hairless, slightly convex stripe separating the callosity from the posterior margin of the ventrite.

***Aedeagus*** paramere as shown in Fig. 46, and endophallus as shown in Fig. 47.

**Female** body (Fig. 5) in average somewhat smaller than in males, length 13.5–15.0 mm, width 6.7–7.8 mm. Punctuation of pronotum sparse and fine, posterior “mirrors” nearly equal is in male (Fig. 15). Traces of striped elytral pattern scarcely visible in posterior portion of elytra. Hind legs shorter than in males, femora not enlarged.

##### Variability.

Most of specimens dorsally present more or less bright blue colour, rarely green, sometimes with bluish reflection, sometimes bicolored, with green pronotum and blueish-green elytra or blue pronotum and violet elytra.

##### Differential diagnosis.

Discriminatory characters with sibling species are provided in Table 1.

##### Type locality.

Turkey, Karaman province, Lale, 10 km south of Karaman.

##### Distribution.

The species has a limited distribution in southern Turkey; it is presently known from the Konya and Karaman provinces (see map in Fig. 43). The reports of *G. festivus* from Adana Province (Polat et al. 2017) may actually refer to this new species.

##### Biological remarks.

*Glaphyrus ciliciensis* sp. nov. inhabits open landscapes in the foothills, primarily dry steppes, as well as ruderal biotopes such as abandoned fields and overgrazed areas overgrown with thorny Asteraceae (Fig. 52). The beetles observed (GS) flying in May, with peak activity occurring from mid-June to the end of June, feeding and mating on blooming *Onopordum* sp. (section *Erecta*) (Fig. 53).

##### Etymology.

The species is named after the ancient region of Cilicia, which is located in an area roughly corresponding to the range of the new species.

#### Glaphyrus (Glaphyrus) neglectus

Taxon classificationAnimaliaColeopteraGlaphyridae

Ghrejan, Kalashian & Sabatinelli sp. nov.

F7859259-4092-5C14-84AB-18CC1BE73BD5

https://zoobank.org/D92A546E-A17D-4730-BF64-26EAB41E283E

Figs 3, 6, 9, 14, 15, 20, 21, 24, 27, 30, 35, 36, 41, 42, 48, 49, 50, 51

Glaphyrus festivus Ménétriés, 1836: Harold 1869: 434 (pars); Champenois 1903: 139, 146 (pars); Reitter 1903: 128 (pars); Zaitzev 1923: 107, 114 (pars); Medvedev 1960: 279 (pars); Iablokoff-Khnzorian 1967: 148 (pars); Baraud 1992: 405, 407 (pars); Nikodým and Bezděk 2006: 100 (pars); Nikodym and Keith 2007: 3 (pars); Nikodým and Bezděk 2016: 95 (pars); Polat et al. 2017: 1509 (pars); Ghahari and Nikodým 2018: 1184 (pars).

##### Type material.

***Holotype*. Armenia** • ♂, Kotayk prov., Jrvezh, 03.07.2017, T. Ghrejyan leg.; IZAY (Fig. 3). ***Paratypes*. Armenia** • 6 ♂♂, 4♀♀, Eriwan; 1898; Korb; MNHN;• 1 ♂; Mt. Ararat; ZIN; • 1 ♂; Erivan; ZIN; • 1 Erivan;• 1 ; Jrvezh; 9.VII.1956; V. Richter; ZIN; • 2 ♀♀; Pashalu, 40 km N of Nakhichevan; 13.VII.1934; Ter-Minasian [Cyrillic script] [Armenia, Vayotsdzor prov., Zaritap] ZIN; • 1 ♂, 1 ♀; Khosrov Reserve;1.VII.1983; A. Kazyuchits; ZIN; • 11 ♂♂; 8 ♀♀; same data as for holotype; IZAY; TGCY; • 10 ♂♂, 9 ♀♀; Ararat prov.; near Urcalanj; 05.VIII.2016; T. Ghrejyan leg.; IZAY; TGCY; • 6 ♂♂, 1 ♀♀, Arménie; Kotayk; Geghadir; VI.2017; J.-L. Alpansèque leg.; COMP; • 30 ♂♂, 9 ♀♀; Kotayk prov.; near Garni; 18.06.2014; T. Ghrejyan leg.; IZAY; TGCY; • 4 ♂♂, Arménie, Kotayk; Garni; 3 km NW; 1480 m; 15.VI.2019; O. Montreuil leg.; COMP; • 2 ♂♂, 1 ♀; TGCY • Vayots Dzor prov.; near Getap; 09.VII.2017; T. Ghrejyan leg.; TGCY; • 3 ♂♂, 1 ♀, Arménie; Ararat; Lanjar; 30.VI.2015; T. Ghrejyan leg; COMP; • 1 ♂, 2 ♀♀; Ararat prov.; Shaghap; 19.VI.2023; P. Hubeny leg.; TGCY • 1 ♀; Armenian SSR [Vayotzdzor prov.]; Yeghegnadzor distr.; env. Elpin; 30.VI.1978; M. Kalashian leg.; MKCY • 2 ♂♂; [Ararat prov.]; “Khosrov Forest” reserve; Vedi area; 29.VI.1978; M. Kalashian leg. (Cyrillic Script); • 2 ♂♂; [Ararat prov.]; Urtsadzor; 1989 m; 23.06.[19]86; S. Bečvář leg. (NMPC); • 2 ♂♂, 1 ♀; [Kotayk prov.]; Garni; 03.07.[19]82; K. Rataj leg. (NMPC); • 2 ♂♂, Chozrovski les; 1200 m. 18.6.79 O. Brodsky leg. (NMPC); • 2 ♂♂; Erivan; Korb [leg.] 1893 (NMPC). **Turkey** • 3 ♂♂, 7 ♀♀; Kars vil.. Karakurt env. 28.VI.1997; M. Malmusi, L. Saltini & L. Padovani leg. (GSCG); • 1 ♂; Van prov. Kiziltas; 1916 m; 38°19'N, 43°14'E; 6.VII.2018; G. Sabatinelli leg. (GSCG); • 6 ♂♂, 8 ♀♀; Van prov. Güzelsu; Bucagi; 2070 m; 28°17N, 43°56'E; 10.VII.2018; G. Sabatinelli leg. (GSCG, DCCE); • 1 ♂, 1 ♀; Van prov.; 2200 m; Kuskum Kiram gec.; 28.VI.2002; G. Sama leg. (GSCG); • 4 ♂♂, 4♀♀; Van prov.; Yetisen; 1800 m; 39°06'N, 43°10'E; 6.VI.2018; leg. G. Sabatinelli (GSCG); • 8 ♂♂, 3♀♀; Van province, Adilcevaz, 20.06.2024, D. Fominykh leg. (AKCM); • 18 ♂♂, 5♀♀, Turquie; Van; Catak; Elmaci; VI.2005; J.-L. Alpansèque leg.; COMP; • 1♂; vil. Bitlis; Guruymak; 1580 m, 25.VI.2002, L. Saltini leg. (GSCG); • 6 ♂♂, 5 ♀♀; Bitlis prov.; Guruymak; 1519 m; 38°33'N, 42°05'E; 9.VII.2018; G. Sabatinelli leg. (GSCG); • 5♂♂, 3♀♀; Van prov.; Dönerdere; Köyü; 1722 m; 38°42'N, 44°08'E; 12.VII.2018; G. Sabatinelli leg. (GSCG); • 1♂; vil. Agri; Patmos; 12.VII.2016; (GSCG); • 4♂♂, 3♀♀; Van prov.; Kurubas gec.; 2239 m; 38°22'N, 43°23'E; 4.VII.2018; G. Sabatinelli leg. (GSCG); • 2♂♂, 1♀; Van prov; Kiziltash; 1916 m; 06.VII.2018; G. Sabatinelli leg. (GSCG); • 3♂♂, 3♀♀; Van prov; Guzelsu Bucagi; 2070 m. 10.VII.2018; G. Sabatinelli leg. (GSCG); • 6♂♂, 1♀; Bitlis prov.; Guroymak 1519 m. 09.07.2018; G. Sabatinelli leg. (GSCG); • 3♂♂, 1♀; Kars prov.; Karakurt. 28.06.-1.07.1997 M. Malmusi, L. Saltini & L. Padovani leg. (GSCG); • 5♂♂, 1♀; Mus prov.; Bulgan Gecidi; 40 km NW Mus; 1640 m; 22-23.VI.99; E.& P. Hajdaj leg. (NMPC); • 2♂♂, 1♀ (MNCP) Bulgan Gecidi; 11.07.2009; J. Haryna leg. (MNCP) • 1♀; Anatolien; Elbistan/Absin; June 1984 (NMPC); • 8♂♂, 3♀♀, Turquie; Lac de Van – Rte de Mus; 08.VII.1959; G. Rémaudière leg.; MNHN; • 1♂, 1♀; Van golu; Timaz env. 28.06.93; V. Malý leg (NMPC); • 3♂♂, 4♀♀; Van prov.; Ossturkey; Kuzgundiran; 2100 m. 04.07.[19]35; Richter leg. (NMPC); • 4♂♂, 1♀; Bitlis prov.; Surphan Dagi; 2500 m; Adilcevaz vil. env. 6.VII.[19]89; B. Brezina leg. (NMPC); • 2♂♂, 2♀♀ (NMPC); Bitlis prov.; 20 km NE Tatvan; 1800 m; J. Hájek & J. Hotovy leg. (NMPC); 1♂, 2♀, Bitlis; MNHN; • 2 ♀♀, Turquie; Bitlis; Tatvan; Asagikolbasi; 1500 m; 10.VII.2003; Staven & Willmer leg.; COMP • 1♂, Turquie; Bitlis; Guroymak; 30.VII.2017; COMP • 1♂; Bitlis prov.; Kultzer; 1912 (NMPC); • 1♂, 1♀ (NMPC) Van prov.; • 1♂, 1♀, Hama Dagh; 2400–3200 m; 03.VIII.1956; H. de Lesse leg.; MNHN; • 1♂ (NMPC) E. Anat. Gevas; Resadiye; 8.7.1973; • 1♀ (NMPC) Hakkari prov.; Akeali; 35 km S. Hakkari; 1700. m; 21.06.2010; W. Grosser leg. (NMPC); • 1♀ (NMPC) Armenien; Erzerum; Reitter (NMPC); • 1♂, 1♀, Erzerum; MNHN; • 1 MNTurquie; Erzerum; E. Reitter leg.; COMP • 1♀ Amasia (h)/ coll. Dr. S. Endrödi (p)/ f; MALATYA, 15.VII.[1]932, leg. V. Aitai (p)/ G. festivus Men. (h) det Endrödi (p)/ D.C. Carlson collection by trade from: (p) S. Endrödi 1977 (h) (DCCE); • 1♀ MALATYA, 15.VII.[1]932, leg. V. Aitai (p)/ G. festivus Men. (h) det Endrödi (p)/ D.C. Carlson collection by trade from: (p) S. Endrödi 1977 (h) (DCCE); • 1♂, Trebizonde; MNHN. **Iran**: • 8 ♂♂, 2♀♀; Iran. Khoi; 1.07.1915; Maljushenco [leg.] IZAY, ZIN. **[? Azerbaijan, Nakhichevan autonomous republic]**. • **1**♂ (NMPC), Araxesthal, Caucasus Leder, Reitter [as shown in Ghrejyan et al. (2023), this record most probably refers to the environs of Ordubad]. • 1 ♂; same data; ZIN.

##### Description.

**Male. *Body*** (Fig. 3) rather large and robust, black, rarely with very faint cobalt-bluish, green, or violet reflections; elytra very rarely brown or reddish-brown. Length: 15.6–17.8 mm (holotype 17.4 mm), width: 7.5–9.0 mm (holotype 8.6 mm).

***Head*** (Fig. 20). Clypeus with widely rounded protruding anterior angles, with a sharp tooth pointing upwards medially. Integument covered with dense, irregular, rough punctures, and sparse, short, brownish hairs. Frons with similar punctuation to clypeus, with rather dense and long blackish setae posteriorly. Fronto-clypeal suture is somewhat obscured by the sculpture but remains visible. First antennomere large, distinctly incurved, and swollen distally, with dense, long dark hairs and short, thick brownish setae. Second antennomere ~3 × shorter, swollen, and covered with dense dark hairs. Antennomeres 3–7 with a single short, delicate seta, and the antennal club has a single short, oblique seta, shorter than those on antennomeres 3–7 (Fig. 14). Dorsal margin of mandibles nearly regularly rounded, without tooth (Fig. 42). Last segment of the maxillary palps distinctly narrowed distally, with truncate apex.

***Pronotum*** (Figs 14, 21) rather convex, with the sides subparallel for approximately the basal two-thirds, leading to obtusely rounded posterior angles, and moderately convergent anteriorly. Anterior margin finely bisinuate with sharp angles and a distinctly detached shiny edging. Posterior margin bisinuate with a protruding medial lobe, and shiny edging separated by a distinct deep groove, which extends slightly forwards along the lateral margins. Disc with dense, rough, partly contiguous punctures; “mirrors”) rather small, with a few punctures. Integument without microreticulation thus rather shiny, covered with dense dark brownish hairs, primarily directed laterally and backwards; without hairs in the glabrous portions. Anterior angles with a tuft of dense, short, thick dark brown setae.

***Scutellum*** small, with fine, somewhat isolated punctures anteriorly, smoother towards the apical part.

***Elytra*** moderately convex, with widely arcuate sides and apices that are widely separated and slightly angularly rounded. The surface “striped,” with each elytron bearing five stripes of more or less dense yellowish setae, partly concealing the surface. Lateral stripe extends from the humeral tubercle but almost does not reach the apex; discal stripes are more or less short and do not reach the elytral base. Integuments between the presutural and second discal stripes covered with dense, rough punctures, while the rest of the surface with sparse, small, and irregular punctures. Lateral margins with a distinct groove that embraces the elytral apices. In lateral view, the margins are only very slightly sinuous (Fig. 9), apical edge of elytra with a clear groove separating convex edge, with row of dense, dark, thick, setae (Fig. 41). Integument without microreticulation, thus rather shiny.

***Pygidium and propygidium***. Dense, short yellowish setae, completely concealing the densely micro-punctate surface.

***Thorax*** with dense, rather rough punctures and long, curve hairs, predominantly dark brown, sometimes mixed with a few yellowish ones. This pubescence is somewhat sparser and shorter medially, while laterally it is denser and sometimes conceals the surface.

***Legs***. Protibiae (Fig. 24) with three large lateral teeth; the edge proximal to them nearly straight but somewhat uneven. Incision between the two distal teeth narrowly arcuate; dorsal surface with a slightly uneven row of punctures bearing long brown hairs and edged by a distinct groove; anteriorly, this row reaches approximately to the level of the incision between the two distal teeth. Mesotibiae (Figs 27, 30) with a slightly uneven outer edge, along which runs a somewhat irregular row of wide flat setae with rounded apices, dorsal surface with a row of rather deep punctures bearing short dark hairs; punctures condensed proximally and sparser distally, reaching approximately to the distal 1/4 of the tibia. Metafemora (Fig. 35) strongly thickened, ~ 1.75 × as long as wide, with a row of distinct rasp-shaped punctures along the middle, bearing short brown setae; proximally and distally, this row expands into areas with similar punctures, which continue along the anterior and posterior margins of the femur; between the medial row and the punctuated margins, the surface is nearly glabrous, with only a few small punctures; large spur of the hind tibia shorter than the first tarsomere; tarsi rather slender; hind trochanter laterally extended into a narrow tooth (Fig. 36).

***Abdomen*** with small, superficial punctures and long yellowish hairs, which almost conceal the integuments. Fifth ventrite with a well detached glabrous callosity. Behind that is a row of thin, short erect hairs situated adjacent to a rather wide, also glabrous and hairless, slightly convex stripe that separates the callosity from the posterior margin of the ventrite.

***Aedeagus***. Paramere see Fig. 48, endophallus see Fig. 49.

**Female**. Body (Fig. 6) length: 14.6–16.4 mm, width: 8.5–9.1 mm. Punctuation on pronotum somewhat sparser and finer than in males, with larger posterior “mirrors” (Fig. 15). Striped elytral pattern is more developed than in males, with wider stripes more extended toward the anterior part. Hind legs short, femora not enlarged.

##### Variability.

Variability very limited; almost all specimens are fully black, with only rare specimens with elytra with very faint blue-cobalt or green or with violet reflections. In rare specimens elytra are brown or reddish-brown.

##### Differential diagnosis.

Discriminatory characters with sibling species are provided in Table 1.

##### Type locality.

Armenia. Kotayk Province, Jrvezh.

##### Distribution.

The species is known from the foothills of the Ararat Plain in Armenia, as well as from Nakhichevan, eastern Turkey, and northwest Iran (see map in Fig. 56).

##### Biological notes.

*Glaphyrus neglectus* sp. nov. inhabits mountain clayey or stony wormwood semi-deserts, occurring at elevations up to 2100 m. In Armenia, the species is active from mid-June to the end of the second decade of July. Both males and females are frequently observed visiting the inflorescences of various Asteraceae, including *Onopordum acanthium*, *O. armenum*, *Carthamus turkestanicus*, *Tomanthea daralaghezica*, and several species of *Cirsium*, *Carduus*, and *Tragopogon* (Figs 50, 51).

##### Etymology.

The specific epithet is derived from the Latin word neglectus, meaning neglected, to highlight the unexpected status of the new species.

## Supplementary Material

XML Treatment for Glaphyrus (Glaphyrus) festivus

XML Treatment for Glaphyrus (Glaphyrus) ciliciensis

XML Treatment for Glaphyrus (Glaphyrus) neglectus

## Additional information

### Use of AI

No use of AI was reported.

### Author ORCIDs

T. Ghrejyan https://orcid.org/0000-0003-4359-2870

M. Kalashian https://orcid.org/0000-0002-2448-9547

G. Sabatinelli https://orcid.org/0000-0001-7197-0619

A. Frolov https://orcid.org/0000-0002-6724-6828

### Data availability

All of the data that support the findings of this study are available in the main text.
